# Dimeric Organization of Blood Coagulation Factor VIII bound to Lipid Nanotubes

**DOI:** 10.1038/srep11212

**Published:** 2015-06-17

**Authors:** Daniela Dalm, Jesus G. Galaz-Montoya, Jaimy L. Miller, Kirill Grushin, Alex Villalobos, Alexey Y. Koyfman, Michael F. Schmid, Svetla Stoilova-McPhie

**Affiliations:** 1 Department of Neuroscience and Cell Biology, University of Texas Medical Branch, Galveston, TX 77555, USA; 2 Department of Biochemistry and Molecular Biology, Baylor College of Medicine, Houston, TX 77030, USA; 3 National Center for Macromolecular Imaging, Baylor College of Medicine, Houston, TX 77030, USA; 4 School of Medicine, University of Texas Medical Branch, Galveston, TX 77555, USA; 5 Department of Pharmacology and Toxicology, University of Texas Medical Branch, Galveston, TX 77555, USA; 6 Sealy Center for Structural Biology and Molecular Biophysics, University of Texas Medical Branch, Galveston, TX 77555, USA

## Abstract

Membrane-bound Factor VIII (FVIII) has a critical function in blood coagulation as the pro-cofactor to the serine-protease Factor IXa (FIXa) in the FVIIIa-FIXa complex assembled on the activated platelet membrane. Defects or deficiency of FVIII cause Hemophilia A, a mild to severe bleeding disorder. Despite existing crystal structures for FVIII, its membrane-bound organization has not been resolved. Here we present the dimeric FVIII membrane-bound structure when bound to lipid nanotubes, as determined by cryo-electron microscopy. By combining the structural information obtained from helical reconstruction and single particle subtomogram averaging at intermediate resolution (15-20 Å), we show unambiguously that FVIII forms dimers on lipid nanotubes. We also demonstrate that the organization of the FVIII membrane-bound domains is consistently different from the crystal structure in solution. The presented results are a critical step towards understanding the mechanism of the FVIIIa-FIXa complex assembly on the activated platelet surface in the propagation phase of blood coagulation.

Factor VIII (FVIII) is an essential cofactor in the blood-clotting cascade. Its importance is illustrated by Hemophilia A, a mild to severe X-chromosome linked bleeding disorder, caused by defective or deficient FVIII^1^,^2^. Factor VIII’s active form (FVIIIa) is the cofactor to the serine protease Factor IXa (FIXa) within the membrane-bound FVIIIa-FIXa complex that catalyzes the activation of Factor X (FX) to Factor Xa (FXa) in the propagation phase of coagulation^3^. Although FVIIIa can bind to FIXa in solution, assembling the FVIIIa-FIXa complex on the activated platelet surface enhances FIXa’s catalytic efficiency more than 100,000 fold^4^,^5^. This significant amplification of FXa generation shows that understanding the membrane-induced structural changes within the FVIIIa-FIXa complex upon membrane binding are physiologically important and of clinical interest.

Factor VIII (FVIII) is a large blood plasma glycoprotein of 2332 amino acids organized in six domains denoted as: A1-A2-B-A3-C1-C2^6^,^7^,^8^,^9^,^10^. The three A domains are homologous to ceruloplasmin^9^,^11^, the B domain is unique, and the two discoidin-like C domains are known to bind to phospholipid membranes^12^,^13^. Purified plasma derived (pd) FVIII exists as a mixture of heterodimers of a heavy chain (HC) comprised of the A1-A2 domains and parts of the B domain and a light chain (LC) of the A1-C1-C2 domains. The LC and HC are non-covalently linked via divalent Ca^2+^ ion(s)^14^,^15^. Factor VIII is activated by Thrombin, resulting in the cleavage of the entire B domain, removal of the LC N-terminal acidic domain and separation of the A2 and A1 domains of the HC^15^,^16^. Activated FVIII (FVIIIa) is a heterotrimer composed of non-covalently linked A1, A2 domains and the LC. The A1 and LC retain the metal ion-dependent linkage through the A1-A3 domains. The A2 to A1 domain association is mediated solely by hydrophobic and electrostatic interactions^4^,^14^,^15^,^17^,^18^,^19^,^20^,^21^. Factor VIIIa is kinetically unstable with a half-life of approximately 6 min *in vitro*, as the A2 domain is only weakly attached to the rest of the molecule (Kd ~500 μM) and dissociates spontaneously^15^,^16^,^17^,^21^,^22^,^23^,^24^. Extensive biochemical and biophysical studies have shown that FVIII membrane binding is mediated through the LC and specifically the C domains^12^,^25^,^26^. The A2 and A3 domains hold the main FIXa binding sites^8^ (Fig. 1).

Human and porcine FVIII expressed in mammalian cells with or without parts of the B domain^27^ have very similar biochemical and hemostatic properties to plasma derived (pd) FVIII and a similar pharmacokinetic profile^2^ when used for treatment of Hemophilia A. Human FVIII lacking the B domain (hFVIII) shows much less structural heterogeneity than pdFVIII and is more suitable for biochemical, biophysical and structural studies^28^. Porcine FVIII lacking the B domain (pFVIII) shares 86% amino acid sequence identity with hFVIII and has similar coagulation activity, forming functional FVIIIa-FIXa complexes with human FIXa *in vivo*^28^,^29^. The pFVIII expression yield is 10 to 14 fold higher than for the human analogue^30^ and its active form (pFVIIIa) is indefinitely stable at concentration greater than 0.3 mM and pH = 6.0, whereas hFVIIIa spontaneously dissociates after only a few minutes *in vitro*^20^,^23^,^31^. These properties of pFVIII are ideal for direct structural studies by cryo-EM in a lipid environment and at close to physiological conditions.

The structure of hFVIII, as organized in 3D crystals in solution (FVIII-3D, Fig. 1A) has been resolved by X-ray crystallography at 3.8–4 Å resolution^19^,^22^,^32^. The three available hFVIII crystal structures are very similar (root mean square deviation between the α-C atoms (RMSD) < 2 Å) and show that the A domains form a triangular heterotrimer stacked on top of the C domains, which are juxtaposed to each other implying that both C domains interact with the membrane^19^,^22^,^32^. From the C2 crystal structure resolved at 1.9 Å^12^, six hydrophobic residues from four adjacent loops have been identified to interact directly with the membrane^13^,^33^. The FVIII-C2 domain membrane-binding interface^12^,^13^ was extended to include amino acid residues from three loops of the C1 domain^22^,^26^,^34^,^35^,^36^(Fig. 1A). We have previously defined the structure of hFVIII-LC helically organized on LNT (3J2S) by fitting the FVIII-LC co-ordinates from the FVIII-3D structure (3CDZ, chain B) within the cryo-EM map of the membrane-bound FVIII-LC-LNT^37^. Modeling of the FVIII-HC co-ordinates from the FVIII-3D structure (3CDZ, chain A) to the FVIII-LC-LNT structure (3J2S) was achieved by aligning the A3 domain from the FVIII crystal structure (FVIII-3D, 3CDZ) to the A3 domain from the FVIII-LC-LNT structure (3J2S) without changing the organization of the A domains’s heterotrimer from the crystal structure (3CDZ)^37^. The hFVIII-LNT structure shown on Fig. 1B is geometrically allowed, as the small hydrophilic C2-A1 and C1-C2 interfaces ( <400 Å^2^), and highly flexible link between the C1 and C2 domains allow for a considerable degree of conformational freedom between the two domains^19^,^22^,^37^,^38^,^39^ (Fig. 1B,C).

The assembly of the FVIIIa-FIXa complex is regulated through high affinity protein-lipid and protein-protein interactions with dissociation constants (Kd) of ~1 nM for each of the FVIII (FVIIIa) and FIXa membrane interactions, and a Kd ~ 15 nM for the FVIIIa-FIXa interaction within the FVIIIa-FIXa complex^40^. The extended FVIIIa-FIXa interaction interface has been identified through a number of functional studies assessing the inhibition by peptides, antibodies, direct mutagenesis, specific point mutations and cross linking studies^41^. The main FVIIIa-FIXa interaction interface is between two loops at the A2 domain surface interacting with two alpha helices from the FIXa-protease domain^42^,^43^,^44^,^45^ followed by an interaction between the FVIIIa-A3 and the FIXa-EGF domains^46^,^47^,^48^,^49^. A fourth interaction site between the FVIIIa-C2 and the FIXa membrane-binding Gla domains has also been identified^49^,^50^(Fig. 1). Based on this knowledge and the FIXa (1PFX) crystal structure^51^ different models of the FVIIIa-FIXa complex have been proposed, none of which satisfies fully the available structural and biochemical information^13^,^22^,^52^. Therefore resolving the FVIII membrane-bound organization is a critical step towards understanding the structural basis of FVIII function and its role in blood hemostasis.

In this work we show the dimeric membrane-bound organization of blood coagulation Factor VIII bound to negatively charged lipid nanotubes (LNT) at close to physiological conditions. We have optimized single bilayer LNT to resemble the activated platelet surface and to bind FVIII with high affinity^37^,^53^,^54^,^55^, thus facilitating our structural and functional studies^38^,^54^,^55^,^56^. The pFVIII-LNT helical reconstruction calculated at 15.5 Å permits us to define the orientation of the FVIII monomers within the membrane-bound dimer. The fitting of the FVIII-LNT structure within the pFVIII-LNT helical map further confirms that the membrane-bound FVIII organization is consistently different from the crystal structure in solution. The dimeric organization of FVIII bound to LNT was also resolved by electron tomography of the same pFVIII-LNT helical tubes followed by subtomograms averaging and single particle tomography (SPT) reconstruction. The symmetry-free and initial model-free pFVIII-LNT SPT structure resolved at 20.5 Å resolution confirmed the relative orientation of the two FVIII molecules within the membrane-bound dimer and the overall FVIII domain organization resolved from the helical reconstruction. Finally, we present a first cryo-SPT structure of pFVIII in solution resolved at 32 Å resolution, showing that pFVIII remains largely globular and monomeric. The presented approach is an effective strategy for structural determination of flexible proteins that can adopt different conformations upon binding to lipid surfaces and/or other proteins to perform a specific function. The blood coagulation Factor VIII is one such protein and defining its structure in a lipid environment is important, as it can lead to modification of the molecule for future clinical trials.

## Results

### Cryo-EM and helical reconstruction of pFVIII-LNT

**Figure 1:**
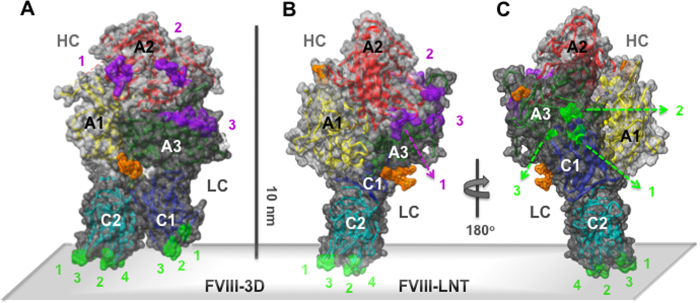
Surface and ribbon presentation of the Factor VIII structure as organized in 3D and membrane-bound helical crystals. **A**. FVIII-3D: Crystal structure of FVIII organized as a 3D crystal in solution (3CDZ)^29^. **B**. FVIII-LNT: Structure of FVIII as organized in a membrane-bound helical crystal on LNT (based on 3J2S), as detailed in^38^. The FVIII light chain (LC) is shown with a dark grey and the heavy chain (HC) – as a light grey surface. The FVIII domains are color coded and shown as ribbons: A1 - yellow, A2 - red, A3 green, C1 - dark blue, C2 - light blue. The membrane surface is shown as a light grey parallelepiped. The FVIII-C1 domain residues: Ile2158-Arg2159 (1), Lys2092-Phe2093 (2), Ile2059 (3) and the FVIII-C2 domain residues: Met119-Phe2200 (1), Leu2251-2252 (2), Val2223 (3) and His2315 (4) proposed to be part of the FVIII - membrane interface are shown as a green surface. The residues from the FVIII-A2 domain: Arg 562 (1) and Lys713 (2), and from the FVIII-A3 domain: Lys1818 (3) part of the FVIIIa-FIXa interface and corresponding loops are shown as a purple surface. The sugar residues NAG: 2234, 2335 and MAN: 2336, 2337, 2338 resolved at the C1-A3 domains’s interface are shown with an orange surface. Both structures are represented with the C2 domain oriented in the same way to the membrane. **C**. The FVIII-LNT structure rotated 180^o^ from **B**. around the y-axis (perpendicular to the membrane surface) showing the position of the C1 domain respective to the C2 domain in the membrane-bound conformation. The scale bar is 100 Å.

**Figure 2:**
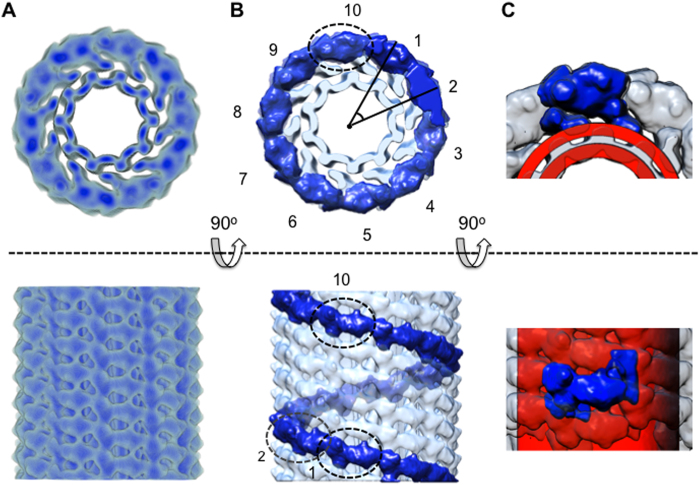
Helical reconstruction of porcine Factor VIII bound to LNT. Orthogonal views along (top row) and perpendicular to (bottom row) the helical (**z**) axis of the final pFVIII-LNT helical reconstruction calculated from 10,430 helical segments and masked to 522 Å length (Supplements 1 and 2). The inner diameter of the helical tube is 200 Å. **A**. Density representation of the pFVIII-LNT helical reconstruction. The maximum density of the pFVIII-LNT helical reconstruction is shown with dark blue, 0.5 (50% of the maximum density level) is shown with medium blue and 0.2 (20% of the maximum density level) is shown with light blue. **B**. Surface representation of the pFVIII-LNT reconstruction drawn at 0.2 contour level (20% of the maximum density). The number of subunits viewed perpendicular to the helical axis is indicated, as well as the azimuthal angle (Δφ) between two adjacent subunits. A single helical strand from the 5 strand pFVIII-LNT helical structure shown on Fig. S3a is highlighted in dark blue. The volume corresponding to one asymmetric unit is indicated with a dashed oval line. **C**. Surface representation of the pFVIII-LNT cryo-EM map (light blue) superimposed with the segmented map corresponding to one asymmetric unit (dark blue). The cryo-EM map corresponding to a bare LNT (red) without attached pFVIII is superimposed with the pFVIII-LNT helical reconstruction to delineate the outer membrane surface of the LNT bilayer.

Previously, we have demonstrated that several FVIII forms organize helically when bound to negatively charged LNT^37^,^55^ and that the asymmetric unit of the human and porcine FVIII helically organized on LNT consists of two FVIII molecules^38^,^56^. To improve the fitting of the known FVIII structures within the cryo-EM map of the membrane-bound FVIII dimer we have calculated a new helical reconstruction of pFVIII-LNT at 15.5 Å resolution. This was achieved by collecting more cryo-EM data of well ordered pFVIII-LNT helical tubes as previously described^55^ and applying a cosine mask to the helical segments to improve the efficiency of the 2D classification process (Supplement 1, Fig. S1). The final pFVIII-LNT helical structure was obtained from 10,430 helical segments (particles set) after 200 consecutive IHRSR refinement cycles (Supplement 2, Fig. S2). The helical parameters converged to a rise Δz = 36.0 Å and azimuthal angle Δϕ  = 35.5^o^, corresponding to a five strand helical structure (Fig. 2A,B Fig. S3). The resolution of the final pFVIII-LNT helical reconstruction was determined to be 15.5 Å at FSC = 0.143 according to the gold standard FSC criterion^57^ (Fig. S3C). The 3D volume of the pFVIII-LNT helical reconstruction was further segmented showing that the membrane-bound pFVIII molecules are organized as dimers within the asymmetric unit (Fig. S3A). To localize the exact position of the membrane bilayer we collected cryo-EM micrographs of LNT alone (with no FVIII attached) with the same lipid composition as the pFVIII-LNT and calculated a 3D structure following the same algorithms as for the pFVIII-LNT reconstruction (Fig. 2C). The volume corresponding to one asymmetric unit, as shown in Fig. 2C is equal to 1,029 × 10^3^ Å^3^ and the surface area to 84.2 × 10^3^ Å^2^. This volume can easily accommodate two FVIII molecules and part of the outer lipid layer of the LNT’s membrane, as the volume and surface area corresponding to the FVIII-3D structure (3CDZ) low-pass filtered to 25 Å are equal to 403.1 × 10^3^ Å^3^ and 31.5 × 10^3^ Å^2^, respectively and the volume and surface area corresponding to the FVIII-LNT structures in Fig. 1B, low-pass filtered to 25 Å are equal to 367.7 × 10^3^ Å^3^ and 31.2 × 10^3^ Å^2^, respectively. Therefore each asymmetric unit in the five strand pFVIII-LNT helical structure can accommodate two FVIII molecules organized as a membrane-bound dimer.

### Single particle tomography (SPT) reconstruction of FVIII bound to LNT

**Figure 3:**
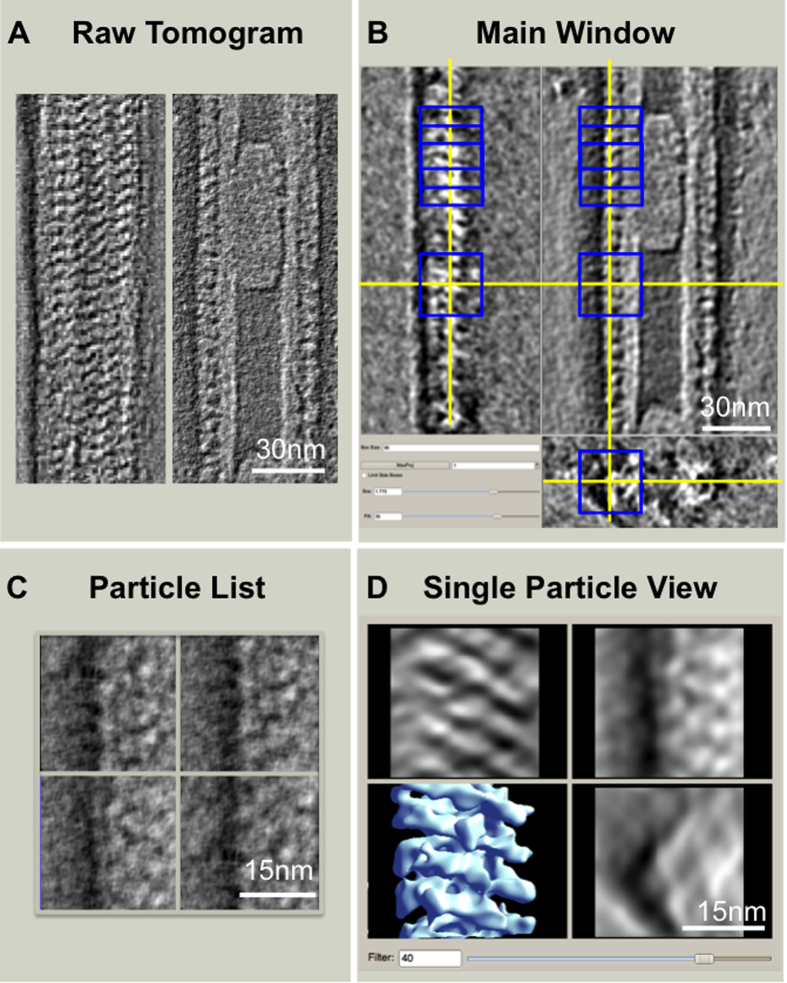
The EMAN2 single particle tomography (SPT) graphical user interface (GUI) and analysis. **A**. Two ~3 Å thick slices from the top (left) and the middle (right) of a negatively stained raw tomogram of a pFVIII-LNT helical tube cut along the helical (z) axis. **B**. Main window of the EMAN2 SPT GUI showing the particle selection (e2spt_boxer.py) window. Three orthogonal slices are shown through the tomogram in A, dynamically low-pass filtered to 50 Å. The blue boxes outline the selected subtomogram volumes at 96 × 96 × 96 voxels (2.9 Å/voxel). The yellow cross denotes the center of the currently selected subtomogram volume shown in D. **C**. Particle List window showing the unfiltered 2D projections in the XY plane for four of the selected sub tomogram volumes in the Main window (B). **D**. Single Particle View window showing three orthogonal projections and the corresponding subtomogram volume (blue surface) currently selected in the Main window (B), which is low-pass filtered to 40 Å before being averaged for the final SPT reconstruction.

**Figure 4:**
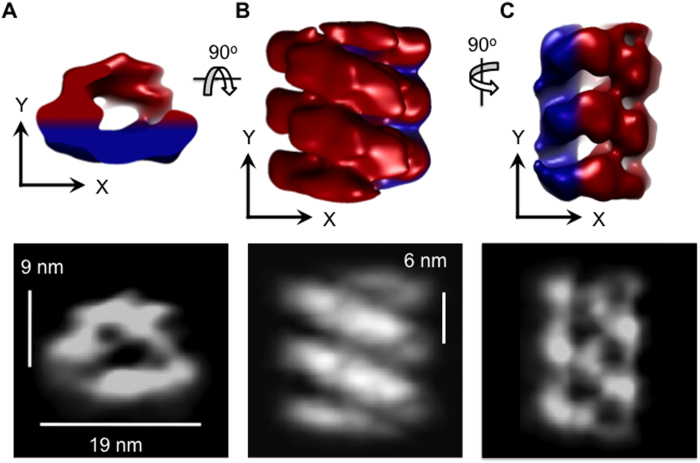
Single Particle Tomography (SPT) reconstruction of porcine Factor VIII bound to LNT. **A**, **B** and **C** are orthogonal views of the pFVIII-LNT SPT reconstruction low pass filtered to 20 Å. **A**. View along the helical (z) axis of the pFVIII-LNT tube. **B**. and **C**. Views perpendicular to the helical (z) axis along the Y and X-axis, respectively. In red is indicated the volume corresponding to the membrane-bound FVIII molecules above the LNT membrane and in blue the volume corresponding to the LNT membrane. The bottom row shows the mass density distribution of the pFVIII-LNT SPT volume along the z, y and x-axis, respectively. Black corresponds to 0 density value and white to the maximum density value of the pFVIII-LNT structure.

FVIII-LNT tubes can be well ordered in the short-range; however they often differ in tube diameter and helical arrangement, which requires of extensive 2D and 3D classification and multiple refinement cycles for helical reconstruction with the IHRSR software (Fig. S1, Fig. S2-I). Imposing symmetry can also raise concerns regarding blurring out important features or giving rise to artifacts. To account for this, we have calculated the structure of pFVIII bound to LNT with a complementary approach for 3D reconstruction based on electron tomography of negatively stained helically ordered pFVIII-LNT tubes^55^,^56^ combined with subtomogram volumes’ averaging and SPT reconstruction (Fig. 3A). The subtomogram volumes’ averaging was performed with the SPT graphical user interface (GUI) implemented in the EMAN2 scientific image-processing suite^58^,^59^ (Fig. 3). The reconstructed pFVIII-LNT tomograms clearly showed the helical arrangement of the membrane-bound molecules on the LNT surface (Fig. 3A). To calculate the pFVIII-LNT SPT structure, 756 subtomogram volumes were extracted from both sides and the top of pFVIII-LNT tomograms, consisting of two adjacent subunits, easily identifiable given the high-contrast of the tomograms (Fig. 3B). The volumes corresponding to two adjacent FVIII molecules bound to the LNT were further aligned and averaged without imposing symmetry and without using any external initial models (Fig. 3D). Having a 3D view (volume) of individual membrane-bound FVIII molecules also facilitated iterative discarding of particles with low correlation scores and helped to isolate a relatively homogeneous population of particles for the final SPT structure. Only 50% of the particles were kept over 24 iterations of alignment. The final membrane-bound, symmetry-free structure of pFVIII bound to LNT was resolved at 20.5 Å according to the gold standard FSC criterion^57^ (Fig. 4, Fig. S4A). The pFVIII-LNT SPT reconstruction showed that along the helical (z) axis each membrane-bound pFVIII helical repeat is ~4 nm wide, spaced ~2 nm apart with a lateral distance of ~6 nm from center to center. Each repeat consisted of two pFVIII molecules, organized as a dimer, forming an arch structure that rises ~9 nm above the LTN membrane (Fig. 4). The pFVIII-LNT SPT volume was further segmented following the same procedure applied to the helical reconstruction (Fig. S4B). The segmented pFVIII-LNT SPT structure shows well-defined densities corresponding to the LNT membrane and the two FVIII molecules within the membrane-bound dimer (Fig. S4B). The volume corresponding to one repeat of the pFVIII-LNT SPT map without the LNT membrane was calculated to be 853.1 ± 19.5 × 10^3^ Å^3^ and can easily accommodate two FVIII molecules (Table S4). The height calculated for the pFVIII dimer above the membrane also exposes the FVIII binding site at the correct height (~80–90 Å) required for the assembly of the FVIIIa-FIXa complex^60^.

### Comparing the FVIII helical and SPT reconstructions

**Figure 5:**
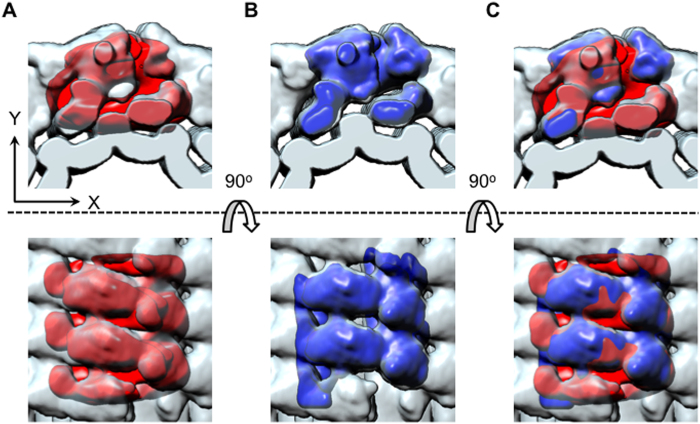
Comparison between the segmented porcine Factor VIII membrane-bound volumes calculated from the SPT and helical reconstructions. The top row presents a view along the helical (z) axis of the pFVIII-LNT tube and the bottom row an orthogonal view perpendicular to helical (z) axis. **A**. The pFVIII-LNT SPT volume (red) is superimposed with the pFVIII-LNT helical reconstruction (light blue). **B**. Two segmented asymmetric units (dark blue) from adjacent helical strands are superimposed with the pFVIII-LNT helical reconstruction (light blue) topographically corresponding to the SPT average. C. Superposition of A. and B.

To compare the pFVIII-LNT SPT and helical reconstructions, first the pFVIII-LNT SPT map of two repeats was aligned to the pFVIII-LNT helical reconstruction (Fig. 5A). Then two asymmetric units (membrane-bound FVIII dimers) from adjacent strands of the pFVIII-LNT helical reconstruction were segmented (Fig. 5B). Comparing the pFVIII-LNT SPT average of two repeats to the helical reconstruction clearly shows that the imposed helical symmetry is correct, as the orientation and spacing of two adjacent repeats from the SPT map, where no symmetry is imposed is the same as those defined in the helical reconstruction (Fig. 5A,B). The differences observed between the two independent pFVIII-LNT 3D reconstructions result mainly from the difference in specimen preparation leading to the flattening of the LNT’s membrane in the pFVIII-LNT SPT reconstruction, due to the adsorption of the pFVIII-LNT tubes on the amorphous carbon surface and slight dehydration from the negative stain. The somewhat lower resolution of the SPT reconstruction also accounts for the fewer details in the pFVIII-LNT SPT map (Fig. 5C).

### Fitting of the FVIII-LNT structure within the membrane-bound pFVIII dimer map

**Figure 6:**
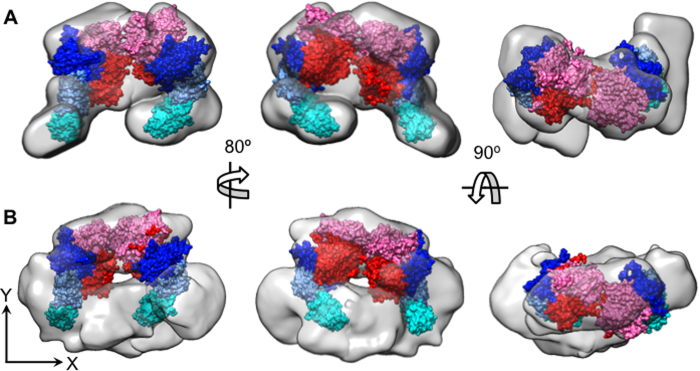
Fitting of the FVIII-LNT structure within the EM map of the membrane-bound pFVIII-LNT dimer segmented from the helical and SPT reconstructions. **A**. Fitting of the FVIII-LNT structure from Fig. 1 to the cryo-EM map of the segmented dimer from the helical reconstruction, as shown on Fig. S5B. **B**. Fitting of the FVIII-LNT structure from Fig. 1 to the EM map of the segmented dimer from the SPT reconstruction as shown on Fig. S5A. The EM maps of the FVIII segmented dimer are shown as a grey surface. The FVIII A1 and A2 domains from the heavy chain are shown in pink and red, respectively. The FVIII A3, C1 and C2 domains from the light chain are shown in blue, light blue and cyan, respectively. The FVIII molecules are oriented in such a way as to interact with the LNT membrane with the identified residues from the apical loops of the C2 domain as shown on Fig. 1B and Fig. S5C.

To resolve the pFVIII molecular orientation within the membrane-bound dimer, the FVIII-LNT and FVIII crystal (FVIII-3D) structures from Fig. 1 were fitted within the pFVIII-LNT helical map with the rigid body docking algorithms implemented in the ‘fit in map’ option of the UCSF Chimera software^61^,^62^ (Fig. 6A). To achieve this the helical pFVIII-LNT map corresponding to the membrane-bound FVIII dimer was further segmented to delineate the densities corresponding to each of the FVIII molecules (Fig. S5B). The FVIII-LNT molecules were then fitted with the rigid body algorithm implemented in the ‘fit to map’ option of UCSF Chimera, starting from different orientations of the FVIII-LNT structures, as detailed in the Materials and Methods section. The best FVIII-LNT structure fit within the segmented membrane-bound FVIII dimer map also allowed for the proper orientation of the C2 domains, such that their membrane-binding loops can interact with the negatively charged LNT membrane (Fig. 6A, Fig. S5B,C). The FVIII crystal (FVIII-3D) structure did not fit as well, as the juxtaposed configuration of the C domains could not be accommodated well in the pFVIII-LNT helical map (Fig. S5Da). The FVIII-LNT structure also fit well in the segmented dimer map from the pFVIII-LNT SPT reconstruction, which was further segmented to delineate the map/density corresponding to each FVIII molecule within the dimer (Fig. 6B, Fig. S5A, Fig. S4B). The lower resolution of the pFVIII-SPT structure resulted in a poorer fit of the FVIII-LNT structure. However, fitting of the FVIII crystal (FVIII-3D) structure within the pFVIII-SPT map left a considerable portion of the FVIII-HC outside of the molecular contour (Fig. S5Db). The orientation of the membrane-bound FVIII molecules obtained from the fitting of the FVIII-LNT structure was such that the main protein-protein interface within the membrane-bound dimer was at the level of the FVIII-heavy chains (HC:A1-A2 domains). This orientation of the FVIII monomers within the membrane-bound dimer does not obstruct the FVIIIa-FIXa interface encompassing the A2 and A3 domains, as shown by the almost identical activity measured for pFVIII in the presence and absence of LNT (Fig. 6, Supplement 6).

### Cryo-electron tomography and SPT reconstruction of pFVIII free in solution

**Figure 7:**
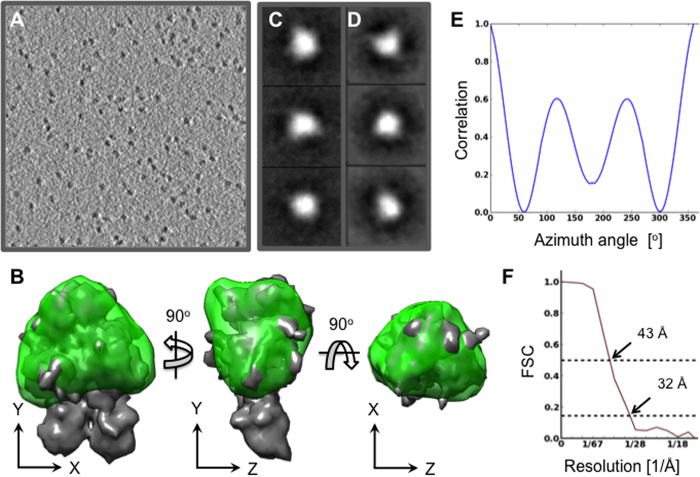
Cryo-ET and SPT reconstruction of porcine Factor VIII free in solution. **A**. A ~4.4 Å thick slice through a cryo-electron tomogram of pFVIII molecules free in solution. **B**. Three orthogonal views of the pFVIII SPT reconstruction (green) superimposed with the FVIII crystal structure (3CDZ) low-pass filtered to 15 Å (grey surface). The z-axis is along the pseudo three-fold axis of the FVIII a domains heterotrimer as resolved in the FVIII-3D structure (3CDZ). **C**. Orthogonal projections and **D**. orthogonal slices through the pFVIII SPT volume. **E**. Rotational self-correlation plot for the FVIII-SPT structure in B. **F**. Gold standard FSC plot for the pFVIII SPT reconstruction showing a resolution of 32 Å at FSC = 0.143 and 43 Å at FSC = 0.5.

To characterize the structure of pFVIII free in solution we collected cryo-ET data using the same buffer and salt conditions as for the pFVIII-LNT cryo-EM samples prepared for the helical reconstruction (Fig. 7). The cryo-electron tomograms of free pFVIII clearly show that FVIII remains monomeric in solution and is well distributed in the thin amorphous ice (Fig. 7A). To calculate the SPT average of pFVIII free in solution, 994 subtomogram volumes of individual pFVIII molecules were extracted from the cryo-electron tomogram (Fig. 7B). The symmetry-free and initial model-free SPT structure of pFVIII not bound to LNT exhibits a predominantly globular shape, the volume of which corresponds to the volume of the three A domains heterotrimer resolved in the FVIII crystal structure (Fig. 7B–D). The rotational self-correlation plot of the symmetry-free and model-free pFVIII SPT reconstruction shows strong pseudo-three-fold symmetry, which agrees well with the FVIII A domains heterotrimer organization resolved in the FVIII crystal structure (Fig. 7E). The resolution of the pFVIII SPT reconstruction free in solution was estimated at 32 Å by the gold standard FSC criterion (Fig. 7F). The C domains, which are known to have multiple orientations in solution^63^,^64^, are not resolved in our SPT average at this resolution and at a surface threshold where the dense, globular head accommodates the crystal structure.

## Discussion

In this study, we have successfully applied single particle tomography (SPT) reconstruction to resolve for the first time the structure of membrane-bound pFVIII helically organized on LNT without enforcing helical symmetry. The pFVIII-LNT SPT reconstruction at 20.5 Å resolution confirms that FVIII organizes as dimers when bound to the LNT, which was previously demonstrated by helical reconstruction^55^,^56^. We are also showing a new pFVIII-LNT helical reconstruction resolved at 15.5 Å resolution. Fitting of the FVIII-LNT structure within the cryo-EM map resolved at 15.5 Å agrees with the concept that pFVIII associates with the membranes mainly through the C2 domain, as previously demonstrated for the pdFVIII organized in membrane-bound 2D crystals^13^ and the hFVIII-LC helically organized on LNT^37^. We can therefore confirm that the membrane-bound pFVIII domain organization on LNT is similar to the one previously resolved from our hFVIII-LC-LNT helical reconstructions^37^. Binding of the whole pFVIII molecules to LNT however shows a new dimeric organization, which was not observed for the LC alone. The orientation of the FVIII molecules within the membrane-bound dimer is ‘head-to-head’, where the FVIII-heavy chains (heads) form the main protein-protein interface and the light chains are oriented further apart, stabilizing the membrane-bound dimer (Fig. 6A). This orientation of the membrane-bound FVIII molecules within the dimer was further confirmed by fitting within the EM map resolved by SPT reconstruction (Fig. 6B).

By simultaneously applying helical and SPT reconstruction approaches to the pFVIII-LNT helical tubes, we have effectively and unambiguously defined the pFVIII membrane-bound oligomeric organization at intermediate resolution (15–25 Å) and close to physiological conditions. This approach compensates for the limitations of each of the helical and SPT reconstruction methodologies if applied separately, which often introduce incorrect helical symmetry and initial volume determination on one hand and stain induced artifacts and missing wedge on the other^65^, that could result in unreliable 3D reconstructions at this resolution. The pFVIII-LNT SPT reconstruction at 20.5 Å confirms that the dimerization is not a result of imposing helical symmetry. The dimeric organization of the membrane-bound pFVIII is also not a result of the helical organization, as it is observed when FVIII is bound to lipid nanodiscs (with the same lipid composition as the LNT) when no helical order is imposed^56^. Finally, the cryo-ET and SPT reconstruction of FVIII free in solution confirms that FVIII remains monomeric in solution, which is consistent with our previously published dynamic light scattering data^28^. Taken together, these facts show that the membrane-bound FVIII dimerization may be critical for its function by providing the macromolecular geometry required for the FVIIIa-FIXa complex assembly on the activated platelet surface. Protein dimerization can be a key factor in the regulation and stabilization of essential proteins for their optimal function^66^, e.g. many enzymes, receptors and transcription factors. In the case of Factor VIII, this membrane-induced dimerization it may stabilize this flexible molecule in conformation, which is optimal for the FVIIIa-FIXa complex assembly and function. Further conformational changes might occur in the membrane-bound Factor VIII domain organization upon binding to the Factor IXa and Factor X on the platelet membrane. These additional conformational changes should not be as extensive as the one observed upon membrane-binding because of the stabilizing role of the membrane for the overall FVIII domain organization. Consequently, the presented FVIII membrane-bound structure can be a critical step towards resolving the structural basis of the FVIII function.

Cryo-EM is an effective approach to define the oligomeric state of macromolecular protein complexes in a membrane environment and at close to physiological conditions. This can be also achieved when the cryo-EM maps are resolved at an intermediate resolution, by applying two complimentary structural approaches such as helical and SPT reconstruction. The membrane-bound pFVIII dimeric organization has evaded previous biochemical and biophysical studies due often to the lack of a membrane and to the ‘sticky’ nature of the protein and its instability in solution. Obtaining such structural information is critical to understand FVIII function and optimize strategies for further high-resolution structure determination, mutagenesis and high-throughput assays required for effective pharmaceutical interventions to regulate blood coagulation at the FVIII level.

The presented work also demonstrates that resolving the structure of membrane-associated proteins at intermediate resolution can be a powerful approach to understand their functional macromolecular organization and can be applied to numerous macromolecular systems that are challenging for high-resolution structure determination by X-ray or cryo-EM. Defining the functional membrane-bound organization at 20 Å resolution is not easily attainable by super resolution light microscopy or cellular electron tomography. In the case of the blood coagulation factors and complexes the presented work offers valuable information towards our understanding of how the activated platelet membrane modulates the clotting process so that it can be amplified and stopped within short time scales.

## Materials and Methods

### Generation of Porcine FVIII-LNT helical assemblies

Porcine FVIII lacking the B domain was expressed in POL 1212 transfected BHK-M cells and purified as previously described^28^.

Galactosylceramide lipid nanotubes containing phosphatidylserine were prepared as described in^37^,^55^. The activity of pFVIII when bound to LNT and when no LNT were present was measured with the one-stage clotting assay - activated partial thromboplastin time (aPTT) test using a Diagnostica Stago clotting instrument (Start® Hemostasis Analyzer, Diagnostica Stago, Inc).

### Cryo-EM sample preparation and data collection

Porcine FVIII-LNT samples were prepared as previously described^67^. Cryo-EM micrographs were recorded with a JEM2100 transmission electron microscope equipped with a LaB_6_ cathode, utilizing a 4 × 4 k Ultrascan CCD camera (Gatan Inc, USA) at a final magnification of 52,000x corresponding to 2.9Å/pix resolution, as previously described^55^.

### Helical reconstruction

The cryo-EM micrographs were processed with the EMAN2 scientific image-processing suite^58^, as previously described^55^. The pFVIII-LNT helical segments were selected by diameter and masked with a cosine mask to create a homogenous data set for the helical reconstruction. The pFVIII-LNT 3D structure was calculated with the Iterative Helical Real Space Reconstruction (IHRSR) algorithm^68^,^69^. The resolution of the final porcine FVIII-LNT reconstruction was evaluated with the Fourier shell correlation algorithms implemented in EMAN2. To achieve this, the final dataset was split into an even and an odd half and 200 IHRSR refinement cycles were carried out with the same parameters as for the final pFVIII-LNT helical reconstruction and a featureless cylinder as initial model.

### Negative stain electron tomography

#### Sample preparation

Negatively stained FVIII-LNT EM grids were prepared as previously described^55^,^56^.

#### Data collection

Data were collected as previously described^55^, with a JEOL2100 electron microscope operated at 200 kV and equipped with a LaB6 cathode. Twelve tilt series were collected. Tilt series were recorded from + /−60^o^ at 2^o^ increments using Serial-EM software^70^, at a target defocus of 2 μm in low-dose mode on a 4 × 4k Gatan Ultrascan CCD camera at an effective magnification of 52,000x corresponding to a sampling of 2.9 Å/pixel using a cumulative dose of ~150–170 e-/Å^2^. The selected tilt series were reconstructed into tomograms with the IMOD software^71^.

### Cryo-electron tomography of pFVIII free in solution

#### Sample preparation

Cryo-EM grids were prepared as previously described^55^.

#### Data collection

Tilt series were collected from + /−60°, at 2° increments using Serial EM^70^, at a target defocus of 6–9 μm in low-dose mode, at an effective magnification of 12,000x corresponding to a sampling of 4.4 Å/pixel and a cumulative dose of 120 e^−^/Å^2^. The images were recorded with a JEOL2200FS electron microscope at 200 KV, equipped with a Field emission gun (FEG) and an energy filter at 20 eV on a DE-12 direct electron camera (3072 × 4096 pixels).

### SPT reconstruction of pFVIII-LNT and FVIII free in solution

All SPT processing was carried out using EMAN2^58^. 756 pFVIII-LNT subtomograms with a box size of 96 × 96 × 96 were extracted from the original raw tomograms to include four-five repeats, using e2spt_boxer.py. The subtomogram were further masked to include two repeats during the alignment process. For the pFVIII free in solution, 994 subtomograms were extracted using a box size of 48 × 48 × 48. All subtomograms were preprocessed for alignment (normalized, Gaussian low-pass filtered and spherically masked); however, once the optimal alignment parameters were found, they were applied to the raw subtomograms for averaging.

The extracted in-solution FVIII sub tomogram volumes were subjected to “all v/s all” alignment (unsupervised hierarchical ascendant classification allowing the merger of unique-best-pairs only, run to convergence through e2spt_hac.py) to create an initial model for subsequent iterative refinement. On the other hand, an initial model for membrane-bound pFVIII sub tomogram volumes was created through a binary-tree averaging approach, and subsequently used for iterative refinement (e2spt_classaverage.py). The sub tomogram volumes were normalized, Gaussian low-pass filtered and spherically masked for alignment purposes. Cross-correlation map normalization was used to avoid missing-wedge bias^72^.

#### FSC correlation curves

The sets of particles used for each SPT reconstruction (pFVIII in solution and pFVIII bound to LNT) were separated into two independent groups after particle picking and processed separately to allow for gold standard FSC computation with EMAN2^58^.

### Fitting of the FVIII-LNT structures within the pFVIII-LNT helical and SPT reconstructions

#### Segmentation of the helical and SPT pFVIII-LNT maps

The final porcine FVIII 3D volume calculated by the helical and SPT reconstructions was segmented with the Seggers watershed segmentation algorithms implemented in the UCSF Chimera Extendible Molecular Modeling System^61^,^73^,^74^ to define the boundaries between individual FVIII molecules helically organized on the LNT surface.

#### Fitting of the segmented asymmetric unit from the helical reconstruction and repetitive unit from the SPT reconstruction within the pFVIII-LNT helical map

All fittings were performed with the “fit in map” options employing the rigid body fitting (docking) algorithms implemented in the UCSF Chimera molecular visualization and analysis software^61^,^62^,^75^

#### Fitting of the FVIII-LNT structure within the helical and SPT pFVIII-LNT maps

The FVIII-LNT structure filtered at 15 Å was fitted within the helical and SPT pFVIII-LNT maps with the same algorithms employed for fitting of the segmented volumes from the same maps. The FVIII-3D (3CDZ) and FVIII-LNT (based on 2S3J) structures with different C-domains organization were fitted (docked) within the cryo-EM map corresponding to the membrane-bound pFVIII-LNT dimer. The FVIII monomer-monomer interface was defined with the ‘find clashes/contact option’ based on standard atom radii and volumes, implemented in the UCSF Chimera Extendible Molecular Modeling System^61^.

### Data deposition

Cryo-EM maps are deposited in the EMDB as: EMD-3026 for the Single Particle Tomography structures and EMD-3027 for the helical structures. The PDB co-ordinates of the fitted structures have been deposited with the EMDB-3027 cryo-EM map (entry EBI-63986).

## Additional Information

**How to cite this article**: Dalm, D. *et al.* Dimeric Organization of Blood Coagulation Factor VIII bound to Lipid Nanotubes. *Sci. Rep.* **5**, 11212; doi: 10.1038/srep11212 (2015).

## Supplementary Material

Supplementary Information
