# Supplementary material for: Dimeric Organization of Blood Coagulation Factor VIII bound to Lipid Nanotubes
Source: Sci Rep. 2015 Jun 17;5:11212. doi: 10.1038/srep11212 (PMC4469981; doi:10.1038/srep11212)
Supplement: Supplementary Information [file srep11212-s1.doc]

**SUPPLEMENTAL INFORMATION**

**Dimeric Organization of Blood Coagulation Factor VIII bound to Lipid Nanotubes.**

(**Membrane-induced dimerization of coagulation Factor VIII)**

Daniela Dalm^1*^, Jesus G. Galaz-Montoya^2,3*^, Jaimy Miller^1^, Kirill Grushin^1^, Alexey Y. Koyfman^4^, Michael F. Schmid^2,3^ and Svetla Stoilova-McPhie^1,5,#^

^1^Department of Neuroscience and Cell Biology, University of Texas Medical Branch, Galveston, TX 77555, USA.

^2^Department of Biochemistry and Molecular Biology, Baylor College of Medicine, Houston, TX 77030, USA.

^3^National Center for Macromolecular Imaging, Baylor College of Medicine, Houston, TX 77030, USA.

^4^Department of Pharmacology and Toxicology, University of Texas Medical Branch, Galveston, TX 77555, USA.

^5^Sealy Center for Structural Biology and Molecular Biophysics, University of Texas Medical Branch, Galveston, TX 77555, USA.

* Equal contribution

#Corresponding author, Tel: +1-979-319-1349; fax: +1-409-747-2200. E-mail address: [svmcphie@utmb.edu](mailto:svmcphie@utmb.edu)

**Supplement 1. 2D analysis of the pFVIII-LNT helical tubes.***

From ~ 2000 recorded cryo-EM micrographs (4096 x 4096 pixels at 2.9 Å/pixel resolution), 554 were selected showing well ordered and straight pFVIII-LNT helical tubes following the algorithms previously described in ref. 56. These micrographs were further filtered, despeckled and inverted in the e2workflow.py graphical user interface (GUI) single particle option provided with the EMAN2 scientific image processing suite^62^ (<http://blake.bcm.edu/emanwiki/EMAN2/Programs>). The pFVIII-LNT helical tubes were boxed at 256 x 256 pixels, 230 pixels overlap with the e2helixboxer.py GUI (EMAN2). The helical segments (particles) sets were corrected for the contrast transfer function of the microscope (CTF) and combined in the single particle reconstruction option of the e2workflow.py interface (e2ctfit.py, EMAN2), as described in ref. 56. With the goal of creating a homogeneous, well ordered final dataset of helical segments, the iterative reference free 2D alignment algorithm (2D-RFA) developed to select particles (helical segments) with similar degree of order and diameter was improved from the one previously described in ref. 56. (**Fig. S1**). After a first round of 2D refinement, only well-ordered particles (helical segments) showing pronounced helical diffraction, were selected for further analysis. In the second round of 2D refinement, only well-ordered particles with similar diameter were selected. The diameter was measured peak-to-peak at the inner layer of the LNT-bilayer in the e2display.py GUI. The helical segments from the final dataset were masked with a rectangular cosine mask of 200 x 180 pixels to account for heterogeneity of the helical order along the same pFVIII-LNT tubular segments. Following this procedure, the homogeneity of the helical segments data set was significantly improved, as illustrated with the improvement of the diffraction patterns shown on **Fig. S1**.

*****The 2D analysis procedure has been used solely to evaluate the quality of the boxed helical segment and to select homogeneous data sets for the helical reconstruction. It has been used separately from the helical analysis procedure shown in Supplement 2.

**
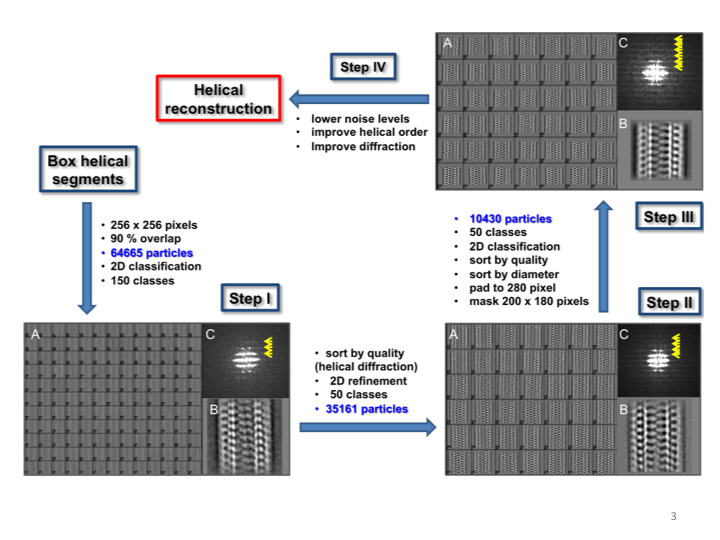
**

**Figure S1. Iterative 2D analysis algorithm for the refinement of the initial helical segments (particles) set. Step I**: Helical segments were boxed at 256 x 256 pixels (2.9 Å/pix) with 90% overlap and a combined data set of 64,665 particles was created in the e2workflow.py GUI (EMAN2)^62^. A 2D reference free alignment (RFA) imposing 150 classes was performed as described in ref. 38 and 56. The particles from class averages showing good helical diffraction were combined and an intermediate set of 35,161 particles was created. **STEP II.** The intermediate set of 35,161 was subjected to a second 2D-RFA classification imposing 50 classes. The class averages were sorted out by inner diameter and the particles with the same diameter were merged in separate particle sets, as previously described in ref. 56. The most populated particles set was of 10,430 particles with inner diameter of 205 ± 5 Å. **STEP III**. A rectangular (200 width x 180 length pixels) cosine mask was applied to each segment of the final particle set of 10,430 helical segments to account for the heterogeneity observed over the length (256 pixels) of the originally boxed helical segments. **A.** Representative subset of the 2D class averages from the 2D-RFA analysis. **B.** Magnified view of the best class average showing the improvement in order and homogeneity due to the exclusion of “bad” particles. **C.** The Fourier transform of **B.** showing the helical diffraction pattern. The improvement of order is illustrated by the increased number of visible layer lines on the Fourier transforms (yellow arrows) from 3 to 6 corresponding to a higher resolution. The third layer line corresponds to a resolution of 1/24 Å and the sixth layer line to a resolution of 1/12 Å.

**Supplement 2. Helical analysis of the pFVIII-LNT helical tubes.***

**Helical 3D reconstruction with the Iterative Helical Real Space Reconstruction (IHRSR) algorithm^58,72^**.

As the pFVIII-LNT helical tubes were often disordered and the helical organization was preserved only over a short length we applied a combination of helical and real space single particle algorithms incorporated in the IHRSR software to calculate the final FVIII-LNT reconstruction. The IHRSR iterations begin with an initial 3D volume of a hollow cylinder with inner and outer diameter calculated from the pFVIII-LNT segments and created in the SPIDER (Shaikh, TR, 2008). After each iteration cycle 90 projections are generated from the consecutive 3D volume 4° apart and used for reference imposed 2D classification ‘multireference alignment’ of the 10,430 particles (ptcls). The helical parameters: Δz (rise) and Δϕ (azymuthal angle) are further applied to the asymmetric 3D volume. The IHRSR cycle (Fig. S2-I) is iterated until convergence of the helical parameter is obtained (Fig. S3B) and no more changes between two consecutive 3D helical volumes are observed.

**Defining the initial helical parameters for the pFVIII-LNT helical 3D reconstruction**

To define the rise (Δz) between two adjacent subunits on one helical strand, we have measured the distance from the equator to the first and second well defined layer line (no 6 and 12) spaced at 72 and 36 Å, respectively on the Incoherent Fourier transform (FFT) of the final 10,430 particles set employed for the IHRSR refinement (Fig. S2-II).

The lack of well defined peaks on the layer lines made it difficult to assign correctly the Bessel order therefore we defined the azymuthal angle (Δϕ) iteratively, as described previously in ref. 56. Only the refinement with initial Δz=36 Å converged and we undertook the search for the azymuthal angle (Δϕ) by rerunning parallel IHRSR refinement with the 10,340 ptcl data set keeping Δz=36 Å and changing Δϕ from 5 to 50° in 5° increments (Fig. S2-III). The search for the optimal azimuthal angle (Δϕ) was initially tested for a 1,000 particles subset for up to 60° as described in ref. 56 and was repeated for the 10,430 particles set to make sure we have correctly defined the azymuthal angle Δϕ=35^o^ from the 1,000 particles subset.

Shaikh TR, Gao H, Baxter WT, Asturias FJ, Boisset N, Leith A & Frank J. 2008 **SPIDER image processing for single-particle reconstruction of biological macromolecules from electron micrographs Nature Protocols, 3. 1941-74**.


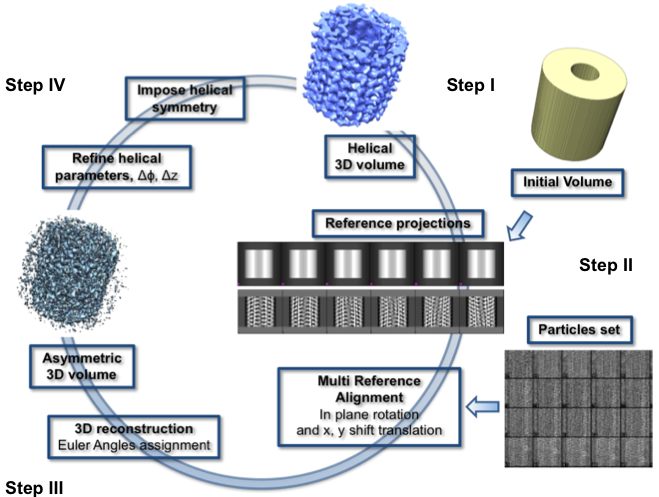


**Figure S2-I. Helical reconstruction with the Iterative Helical Real Space Reconstruction (IHRSR) algorithm**. **Step I.** An initial volume of a hollow cylinder is created, with inner and outer diameter corresponding to the pFVIII-LNT tubes and 90 reference projections, 4° apart are created. **Step II.** Reference imposed 2D classification of the 10,430 particles is carried out with the initial projections and then consecutive 3D volumes. **STEP III**. An asymmetric 3D volume is generated from the class averages. **STEP IV.** The helical parameters (Δϕ and Δz) are applied to the asymmetric 3D volume to calculate the final 3D reconstruction. The ‘Reference projections’ shown on Fig. S2-I are a representative subset of the 90 projections. The reference projections on the top row are from the initial 3D volume and on the bottom row from the final pFVIII-LNT volume (after 200 refinement cycles). The IHRSR cycle is iterated until convergence of the helical parameters is obtained (Fig. S3B) and no more changes between two consecutive 3D helical volumes are observed.


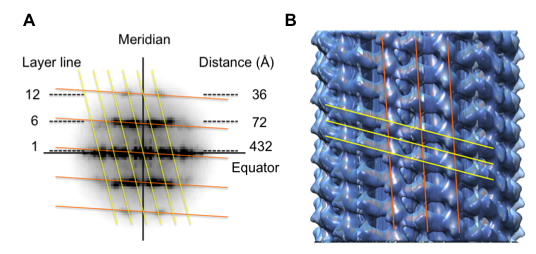


**Figure S2-II. A. Incoherent FFT of the 10,430 particles data set. The layer lines number is indicated on the left and the distance from the equator on the right.** The 2D lattice is indicated with yellow and orange lines. **B.** The 2D lattice in reciprocal space (Fourier space) is rotate by 90° and flipped horizontally to be superimposed with the pFVIII-LNT helical reconstruction from 10,430 particles, showing that the imposed initial helical parameters (Δz=36 Å and Δϕ=35^o)^ and C5 rotational symmetry yield a correct 3D helical reconstruction with the IHRSR algorithms.


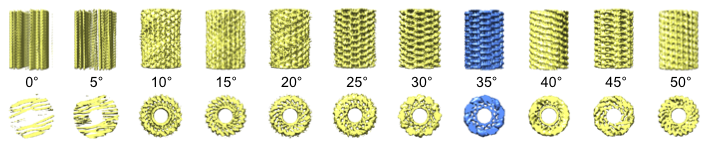


**Figure S2-III.**  3D helical reconstructions of pFVIII-LNT from the same data set of 10,430 helical segments, imposing initial Δz = 36 Å and changing the azimuthal angle Δϕ from 5 to 50° in 5° increment. Each IHRSR refinement was carried out for 200 cycles.

**Supplement 3. Segmentation of the pFVIII-LNT helical reconstruction**


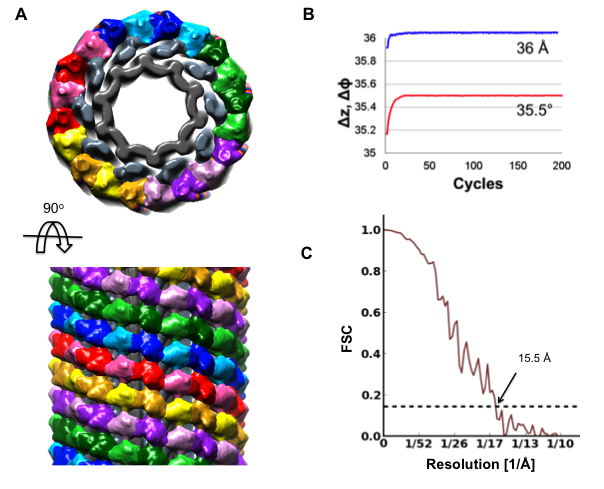


**Figure S3. A.** Views along (top) and perpendicular to (bottom) the helical (**z**) axis of the final porcine FVIII-LNT 3D volume calculated from 10,430 helical segments masked to 522 Å (180 pixels) length as shown in Supplements 1 and 2. The five strands of the pFVIII-LNT helical structure are color-coded. The segments/volumes corresponding to the individual FVIII molecules within one asymmetric unit (dimer) forming the five helices are colored in light and dark shades, respectively. The molecules at the edge of the pFVIII-LNT tube are removed for clarity. The density corresponding to the inner LNT monolayer is colored in dark grey and the density corresponding to the outer LNT monolayer including the membrane-binding part of the molecule is colored in grey. **B.** Convergence plot of the helical parameters Δϕ=35.5° and Δz=36.0 Å over 200 IHRSR refinement cycles as described in Supplement 2. **C.** Fourier shell correlation (FSC) plot between two independent porcine FVIII-LNT helical reconstructions showing a resolution of 15.5 Å at FSC=0.143.

**Supplement 4. Segmentation of the pFVIII-LNT SPT reconstruction**


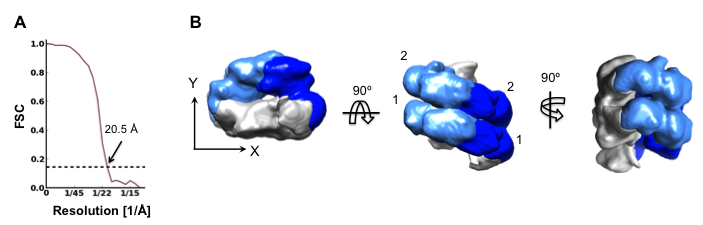


**Figure S4. A.** Gold standard FSC curve for the FVIII-LNT SPT 3D reconstruction shown in Fig. 4, showing a resolution of 20.5 Å at FSC=0.143 and 23.5 at FSC=0.5. **B.** Segmentation of the FVIII-LNT SPT volume. The volumes corresponding to partial molecules at the edge have been removed. The volume corresponding to the LNT membrane is colored in grey and the densities corresponding to the membrane-bound FVIII molecules are colored in dark and light blue, respectively. **B.** Volumes and area of the segmented SPT volume colored light and dark blue, respectively, as calculated with the UCSF Chimera software (65) Volume options (66).


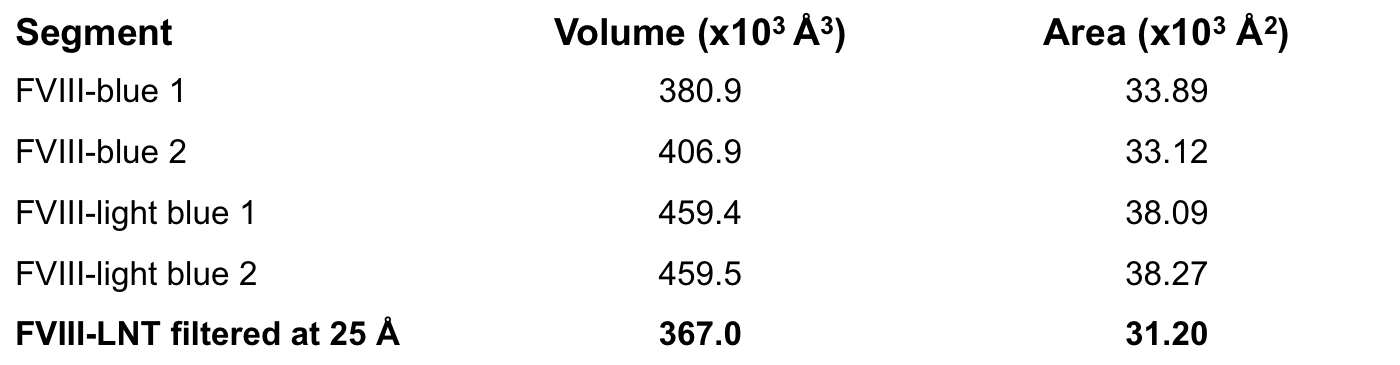


**Table S4.** Volume and area corresponding to the segmented FVIII monomers as calculated with the UCSF Chimer ‘Volume data’ option from the pFVIII-LNT SPT reconstruction, as shown on Fig. S4B.

**Supplement 5. Segmentation of the membrane-bound pFVIII dimer and fitting of the FVIII-LNT and FVIII-3D structures.**

**
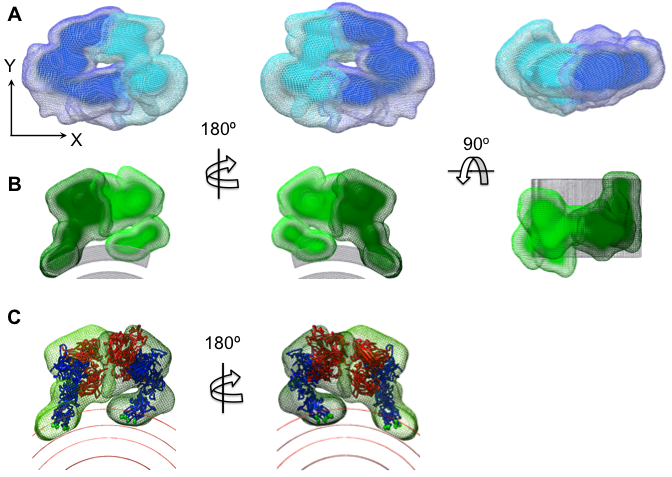
**

**Figure S5. A.** Segmentation of the pFVIII-LNT SPT reconstruction corresponding to the repetitive unit (membrane-bound FVIII dimer). The surface (mesh) and density (solid color) corresponding to each FVIII molecule with part of the LNT membrane are shown in a light and dark shade of blue. **B.** Segmentation of the pFVIII-LNT helical reconstruction corresponding to one asymmetric unit (membrane-bound FVIII dimer). The map surface (mesh) and density (solid color) corresponding to each FVIII molecule is presented with a different shade of green. The map corresponding to the LNT membrane is shown as a grey mesh. **C.** Fitting of the FVIII-LNT structure within the membrane-bound FVIII dimer map from the helical reconstruction. The FVIII - light chain is shown with blue ribbons and the FVIII - heavy chain with red ribbons. The C2 domain membrane-binding residues are shown as green spheres. The contour corresponding to the LNT bilayer is shown with red-purple lines.


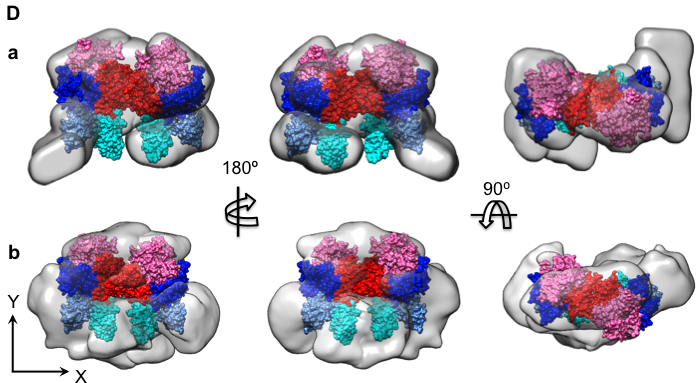


**Figure S5. D.** Fitting of the FVIII crystal structure (FVIII-3D, Fig. 1) within the segmented maps of the dimer from the pFVIII-LNT helical (a) and SPT (b) reconstructions shown as a grey surface. The FVIII A1 and A2 domains from the heavy chain are shown in pink and red, respectively. The FVIII A3, C1 and C2 domains from the light chain are shown in blue, light blue and cyan, respectively**.** The FVIII molecules are oriented in such a way, as to interact with the LNT membrane with the identified residues from the apical loops of the C2 domain as shown on Fig. 1B and Fig. S5C.

**Supplement 6. Clotting activity of pFVIII.**

The pFVIII-LNT samples for the aPTT (activated partial thromboplastin time) activity tests were prepared as follow. The LNT and FVIII were prepared as described in Materials and Methods. A volume of 2 µl pFVIII (1 mg/ml) was mixed with 20 µl LNT (1mg/ml, 10x excess). Samples were diluted for the activity to fall within the standard curve, incubated in FVIII deficient plasma (<1% activity, George King Bio-Medical, Inc.) with aPTT reagent (TriniCLOT Automated aPTT, Tcoag Ireland Limited, Ireland) for 4 min at 37°C. The activity was evaluated against Factor Assay Control Plasma (FACT, 1 U/ml of FVIII activity) (George King Bio-Medical, Inc.)


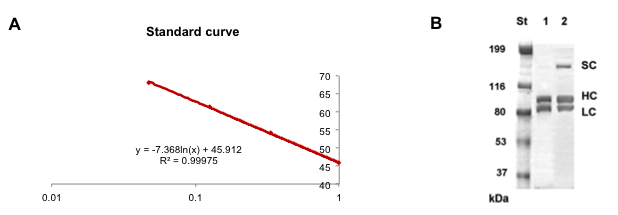


**Figure S6. A. Standard curve for porcine FVIII clotting activity.** The standard curve is calculated for Factor Assay Control plasma (FACT) containing 1 U/ml human FVIII (plasma derived). The common logarithm of the FVIII concentration in (U/ml) is plotted on the x-axis. The clotting times in seconds (s) were measured for the following FACT dilutions: undiluted, 0.33, 0.125 and 0.0476, and plotted on the y-axis, as described following the protocols in Duffy et al. 1992. **B.** SDS-PAGE of recombinant human FVIII (1) and porcine FVIII (2). The bands corresponding to the FVIII single chain (SC), light (LC) and heavy (HC) chains are indicated. Molecular weights of standard proteins are indicated in the lane labeled St.


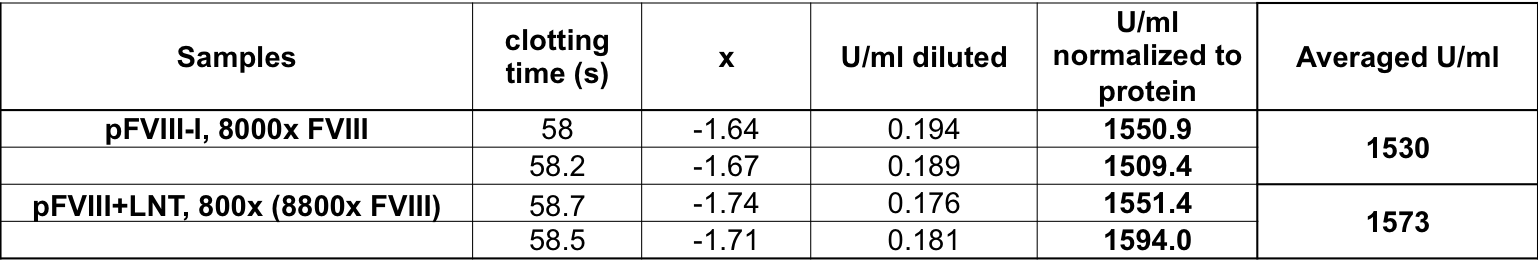


**Table S6. Activity of porcine FVIII when bound to LNT and when no LNT are present, as obtained with the aPTT test.** The porcine FVIII activity is measured in FVIII deficient plasma in the presence and absence of LNT. The porcine FVIII concentration was normalized.

Duffy, E.J. and P. Lollar, *Intrinsic pathway activation of factor X and its activation peptide-deficient derivative, factor Xdes-143-191.* J Biol Chem, 1992. **267**(11): p. 7821-7.
